# Exploiting venom toxins in paratransgenesis to prevent mosquito-borne disease

**DOI:** 10.1186/s13071-025-06663-9

**Published:** 2025-01-29

**Authors:** Stephanie French, Rachael Da Silva, Janet Storm, Christida E. Wastika, India Cullen, Martijn ten Have, Grant L. Hughes, Cassandra M. Modahl

**Affiliations:** 1 Centre for Snakebite Research and Interventions, Liverpool School of Tropical Medicine, Liverpool, UK; 2 Departments of Vector Biology and Tropical Disease Biology, Centre for Neglected Tropical Disease, Liverpool School of Tropical Medicine, Liverpool, UK

**Keywords:** Rift Valley fever virus, Yellow fever virus, Japanese encephalitis virus, West Nile virus, Chikungunya virus, Dengue virus, Zika virus, Snake, Scorpion, Spider, *Aedes*, *Anopheles*

## Abstract

Mosquitoes are responsible for the transmission of numerous pathogens, including *Plasmodium* parasites, arboviruses and filarial worms. They pose a significant risk to public health with over 200 million cases of malaria per annum and approximately 4 billion people at risk of arthropod-borne viruses (arboviruses). Mosquito populations are geographically expanding into temperate regions and their distribution is predicted to continue increasing. Mosquito symbionts, including fungi, bacteria and viruses, have desirable traits for mosquito disease control including spreading horizontally and vertically through mosquito populations and potentially colonising multiple important vector species. Paratransgenesis, genetic modification of mosquito symbionts with effectors to target the pathogen rather than the vector, is a promising strategy to prevent the spread of mosquito-borne diseases. A variety of effectors can be expressed but venom toxins are excellent effector candidates because they are target specific, potent and stable. However, the only toxins to be explored in mosquito paratransgenesis to date are scorpine and mutated phospholipase A2. To enhance the scope, effectiveness and durability of paratransgenesis, an expanded arsenal of effectors is required. This review discusses other potential toxin effectors for future paratransgenesis studies based on prior in vitro and in vivo antiparasitic and antiviral studies and highlights the need for further research and investment in this area. In terms of mosquito-borne diseases, paratransgenesis strategies have been developed to target *Plasmodium*. We postulate the potential to apply this principle to target arboviruses using antiviral toxin effectors.

**Graphical Abstract:**

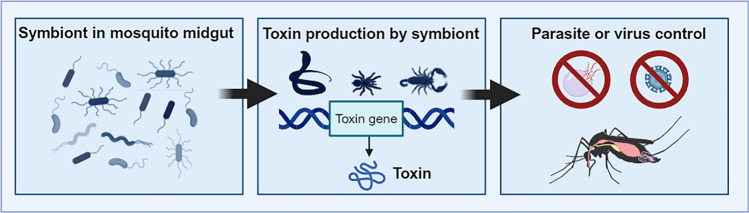

**Supplementary Information:**

The online version contains supplementary material available at 10.1186/s13071-025-06663-9.

## Background

Mosquito-borne pathogens such as parasites and arthropod-borne viruses (arboviruses) pose a significant risk to public health [1]. Malaria is one of the most common parasitic diseases globally with an estimated 249 million cases and 608,000 deaths reported in 2022, mostly in children under 5 years in sub-Saharan Africa [2]. Over half the world’s population is at risk of infection by arboviruses, including Rift Valley fever virus (RVFV), yellow fever virus (YFV), Zika virus (ZIKV), Japanese encephalitis virus (JEV), West Nile virus (WNV), dengue virus (DENV) and chikungunya virus (CHIKV) [3].

Due to climate change and other anthropogenic factors, the burden of mosquito-borne diseases is intensifying [4–6]. There is no single solution for the control of mosquito-borne disease and multiple strategies are required. This multi-pronged approach will require location-specific strategies influenced by environmental and economic factors, governing bodies and disease prevalence [7]. In this regard, novel strategies and tools are urgently required to develop an integrated control strategy.

Paratransgenesis, the genetic engineering of symbionts with anti-pathogenic effectors to control disease transmission, represents a potentially promising strategy. The technique was originally developed by Beard et al. to control *Rhodnius* *prolixus* (triatomine/kissing bug) from spreading the causal parasite of Chagas disease (*Trypanosoma* *cruzi*) [8]. A gram-positive bacteria, *Rhodococcus* *rhodnii*, that occurs at high concentrations within the hindgut of *R.* *prolixus* was genetically engineered to express a trypanocidal immune peptide, Cecropin A. This resulted in a decreased *T.* *cruzi* infection rate in *R.* *prolixus* and was approved as an integrated pest management program in South and Central America [8].

Paratransgenesis offers several advantages. It is scalable because transgenic microorganisms can be grown to large quantities at low cost [9, 10]. The technique is not limited to single mosquito species because symbiotics can potentially colonise multiple important vector species [9, 10]. Moreover, the symbiont can be maintained within the ecosystem by vertical, horizontal and trans-stadial transmission, mitigating the need for re-introduction [9, 10]. Finally, and perhaps most importantly, it is a manipulable system that can be altered to target different pathogens or keep pace with resistance by exploiting different effectors. As such, the discovery and development of novel anti-pathogen molecules are critical for paratransgenesis implementation.

Venom toxins are excellent candidates for effectors in paratransgenesis. Venoms are complex mixtures of toxic proteins, peptides and small molecules delivered through the infliction of a wound from a bite or sting [11, 12]. Venoms of hymenopteran insects such as bees and wasps are diverse, consisting of peptides, enzymes and neurotransmitters [13], whilst scorpion and spider venoms largely consist of neurotoxins, which modulate a variety of channels including voltage-gated potassium, sodium and calcium ion channels, acid-sensing ion channels, calcium-activated potassium channels, glutamate receptors and glutamate transporters [14, 15]. Snake venom consists of haemotoxins, cytotoxins and neurotoxins that can be grouped into superfamilies by structure, with snake venom phospholipase A2s (PLA_2_s), metalloproteinases, serine proteinases and three-finger toxins being the most abundant [16]. Venom toxins have high specificity, potency and stability [11] and are less susceptible to bioaccumulation than chemical insecticides [17]. The venoms of many hymenopteran insects, scorpions, spiders and snakes have been studied for their potential antiparasitic [18–20] and antiviral [21–24] properties. This review highlights the untapped potential of venom toxins as effectors in paratransgenesis. We discuss successful paratransgenesis studies that have been undertaken with antimalarial venom toxins as proof of principle and the need for specific screening of venom toxins to identify effectors is highlighted. Regarding mosquito-borne diseases, paratransgenesis strategies have focused on targeting *Plasmodium*, the causal agent of malaria. However, we suggest that paratransgenesis could be applicable to target arboviruses through the use of antiviral venom toxins.

### Antiparasitic venom toxins as effectors to target *Plasmodium*: current position

**Fig. 1:**
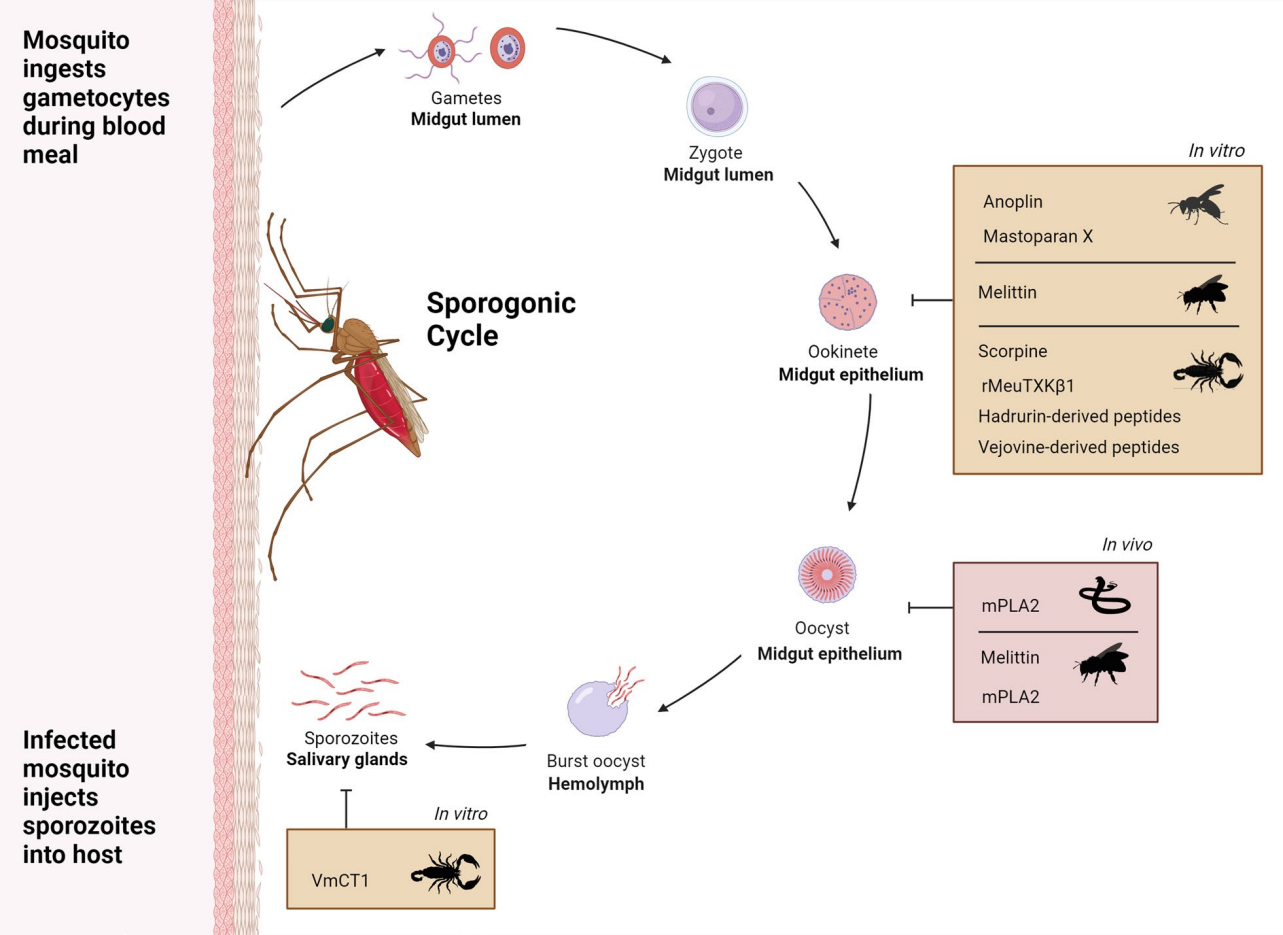
Overview of in vitro and in vivo studies showing activity of toxins against the mosquito stages of *Plasmodium* sp. Venom toxins have shown anti-plasmodial activity within mosquitoes (in vivo, pink panel) and anti-plasmodial activity when added directly to isolated *Plasmodium* stages (in vitro, orange panels). The cycle shows the developmental stages of *Plasmodium* sp. within the mosquito vector and inhibitory arrows indicate the stage targeted by the venom toxin. *Plasmodium* male and female gametocytes, the sexual stages that differentiate into gametes, are taken up by the mosquito in a blood meal and merge to form a zygote which develops into a motile ookinete. The ookinetes invade the epithelial lining of the mosquito midgut and differentiate into oocysts that generate sporozoites which are released into the haemolymph, invade the salivary gland and are injected in the skin of the next host during a blood meal

Previous mosquito paratransgenesis strategies have focussed on targeting *Plasmodium*, the causal parasite of malaria. The species of *Plasmodium* responsible for causing malaria in humans are *Plasmodium* *falciparum*, *P.* *vivax,* *P.* *ovale,* *P.* *knowlesi* and *P.* *malariae*, with the former being responsible for > 90% of malaria deaths [2]. Mosquitoes from the *Anopheles* genus are responsible for the transmission of malaria. Paratransgenesis targeting *Plasmodium* must use effectors that inhibit the parasite stages within the mosquito: gametes, ookinetes, oocysts or sporozoites (Fig. 1) [25]. Two venom toxins have effectively been utilised as effectors (Table 1): scorpine, an excitatory neurotoxin from *Pandinus* *imperator* with antibacterial and antiparasitic properties, and mPLA_2_, a PLA_2_ from bee venom with a point mutation (H67N) to prevent enzyme activity and toxicity to bacteria. mPLA_2_ expressed in *Escherichia* *coli* induced a moderate reduction of oocyst numbers from *Plasmodium* *berghei*, a rodent malaria model, when fed to *Anopheles* *stephensi*. However, the bacterium survived poorly in the mosquito [26]. Scorpine and mPLA_2_ expressed in *Serratia* [27] and *Plasmodium* *agglomerans* [28] were able to effectively colonise the midgut of *Anopheles* *gambiae* and decreased the number of *P.* *falciparum* oocytes in infected mosquitoes. Scorpine has also been expressed in *Asaia* [29], a bacteria found in *Anopheles* *sp.*, *Aedes* *aegypti* and *Ae.* *albopictus* [30–36] that is transmitted vertically, horizontally and transstadially [31], and *Metarhizium* *anisopliae*, a fungus pathogenic to adult mosquitoes that infect through direct contact with the cuticle [37]. Transgenic *Asaia* expressing scorpine significantly reduced the number of *P.* *berghei* oocytes in the mosquito midgut; however, constitutive expression of the toxin compromised bacterial fitness. To improve bacterial fitness, blood meal-inducible promoters within the mosquito microbiome were identified and used to conditionally express scorpine. This enabled *Asaia* to maintain fitness and compete with wild-type *Asaia*, whilst oocyst midgut numbers in *A.* *stephensi* decreased by ~ 90% and prevalence decreased by up to 20%, indicating a decrease in infection potential [38]. Transgenic *M.* *anisopliae* expressing scorpine inhibited sporozoite invasion of the salivary glands by 90% compared to control mosquitoes without *M.* *anisopliae* [37].

**Table 1:** Summary of the toxin effectors used in paratransgenesis studies that target *Plasmodium*

Venom effector	Mosquito species	Vector symbiont	Effect	Refs.
Scorpine	*Anopheles* *stephensi*	*Asaia* *bogorensis*	63% reduction in oocyst number of *Plasmodium* *berghei*	[29]
Scorpine	*An.* *stephensi*	*Asaia* *bogorensis*	90% reduction in oocyst number of *Plasmodium* *berghei*	[38]
mPLA_2_	*An.* *stephensi*	*Escherichia* *coli*	23% reduction in oocyst number of *Plasmodium* *berghei*	[26]
Scorpine and mPLA_2_	*An.gambiae* *An.* *stephensi*	*Pantoea* *agglomerans*	98% reduction (scorpine) and 85% reduction (mPLA_2_) in oocyst number of *Plasmodium* *falciparum*	[28]
Scorpine and mPLA_2_	*An.* *gambiae*	*Serratia* *marcescens*	93% reduction (scorpine) and 86% reduction (mPLA_2_) in oocyst number of *Plasmodium* *falciparum*	[27]
Scorpine	*An.* *gambiae*	*Metarhizium* *anisopliae*^a^	90% reduction in sporozoite invasion of salivary glands	[37]

*mPLA*_*2*_ inactive mutant (H67N) PLA_2_

^a^Not traditional paratransgenesis as defined by Ratcliffe et al*.* [10] given *Metarhizium* *anisopliae* is a mosquito parasite

Despite promising preliminary mosquito paratransgenesis data, only a limited number of effector molecules have been assessed with mPLA_2_, with scorpine being the only venom toxin effector that has been experimentally tested in mosquito paratransgenesis. An expanded arsenal of molecules is required to allow a multi-faceted and adaptable approach to paratransgenesis. Importantly, expression of multiple effectors has been shown to enhance efficacy [27, 28] and can enable several stages of the pathogen life cycle to be targeted, increasing robustness. The risk of resistance development can be reduced through identification and use of multiple effectors with different mechanisms of actions and/or broad-spectrum actions. There is also potential to target multiple pathogens through co-expression of effectors or use of effectors with multiple mechanisms of action. Finally, it is important to have a diverse effector library available to mitigate resistance and enable new paratransgenesis replacement strategies.

Venom toxins have the potential to act as effectors due to their antiparasitic activity (Fig. 1, Additional file 1: Table S1). However, most of these studies have focussed on the intraerythrocytic asexual stages of *Plasmodium* within the mammalian host [19], in line with research more applicable to the identification of antimalaria therapeutics. Few studies have screened toxins to identify effectors for paratransgenesis, but for effectors to be useful they must target the *Plasmodium* stages occurring in the mosquito [25].

Several α-helical linear peptides such as anoplin and mastoparan-X from wasp venom [39], melittin from European honeybee venom [39] and MeuTXKβ [40] from *Mesobuthus* scorpion venom inhibit ookinete development. Another linear helical peptide, specifically scorpion toxin VmCT1 from *Vaejovis* *mexicanus*, is effective in vitro against *Plasmodium* *gallinaceum* sporozoites, a poultry model of the last stage of *Plasmodium* development within the mosquito [41]. Antimicrobial peptides from scorpions including scorpine and synthetic peptides based on vejovine and hadrurin also inhibit ookinete development in vitro [42–44]. In vivo studies have found PLA_2_ derived from the venom of the rattlesnake *Crotalus* *adamanteus* reduced the number of oocysts by 99% when mixed with cultured *P.* *falciparum* gametocytes and fed to *An.* *gambiae* or *An.* *stephensi* mosquitoes [45]. A similar reduction in *P.* *gallinaceum* oocyst number in *Ae.* *aegypti* was achieved. Interestingly, the PLA_2_ toxin did not affect ookinete viability but acted on the midgut surface, preventing ookinete maturation to oocytes. A similar effect was observed for a PLA_2_ from bee venom in *Aedes* *fluviatilis* [46] and melittin [39].

### Antiparasitic venom toxins as effectors to target *Plasmodium*: future studies

Ookinete to oocyst development in the midgut, the bottleneck of malaria transmission [47], is the best target for effector screening. Future studies should focus on *P.* *falciparum*, the species primarily infecting humans and causing most malaria deaths. Although experiments with *P.* *falciparum* are challenging and limited to in vitro culture of the intraerythrocytic stages of the parasite, ookinetes can be generated in vitro by gametocyte differentiation in specialised medium enabling toxins to be rapidly screened [48]. However, studies have mainly been performed with *P.* *berghei* and *P.* *gallineceum*, species that infect rodents and poultry, respectively, as described above [39–46]. This is because they can be maintained as intraerythrocytic stages in mice or chickens to generate a high density of gametocytes [49], the sexual stage required for oocyst development within the mosquito.

An ideal high-throughput pipeline would involve the screening of toxins on ookinete development in vitro with successful candidates taken forward to in vivo development of *Plasmodium* in the mosquito, as described by Carter et al., 2013 [39]. Using multiple toxins could potentially help prevent resistance and can have synergistic effects [37]. Fang et al. showed that co-expression of scorpine with either eight copies of salivary gland and midgut peptide 1 (SM1) or a single-chain antibody that binds *P. falciparum* (PfNPNA-1) reduced *Plasmodium* sporozoite count to a greater extent than either of the three peptides alone. Therefore, toxins that pass initial screening should be screened in combination with known anti-*Plasmodium* peptides.

The compatibility of the toxins with mosquito symbionts must also be assessed, with particular focus on symbionts such as *Asaia* *bogorensis*, *Serratia* *marcescens* and *M.* *anisopliae*, which have yielded promising results in paratransgenesis studies. Toxicity towards specific bacteria and fungi can be tested relatively easily by carrying out minimum inhibitory concentration assays. *Asaia* warrants particular attention because it is an excellent candidate for paratransgenesis as it colonises the midgut of a range of mosquitoes, including *Anopheles* sp., *Aedes* *aegypti* and *Ae.* *albopictus* [30, 31, 34, 35, 50, 51], and is transmitted vertically and horizontally [31, 32]. *Asaia* also contains known blood meal-inducible promoters that have been validated in a scorpine secretion plasmid [38].

In addition, assays must be undertaken to ensure the toxin is specific for the pathogen. This should involve assessing any potential effect on mosquito fitness [39]. Assays include basic in vitro cell viability assays on mosquito cell lines and in vivo studies evaluating the effect of toxin feeds on mosquito longevity and fecundity. Maintained longevity and fecundity are important to ensure the symbiont spreads effectively throughout the mosquito population, a critical determinant of paratransgenesis success. The impact of toxins on mosquito behaviour should also analysed, such as biting and mating frequency, because these can affect disease transmission. This has previously been tested downstream for wild-type and recombinant *Serratia* strains expressing antimalarial effectors [27]; however, additional upstream testing would allow toxins to be down-selected. Nevertheless, the importance of also undertaking the above studies with transformed symbionts cannot be overstated.

From an ecological and safety perspective, it is important to assess potential off-target effects because of the possibility of transgenic symbionts colonising other species. Toxicity of toxin peptides towards pollinator species should be evaluated by in vivo feeding assays, and mammalian toxicity should also be assessed using in vivo and in vitro assays. Again, these studies must also be conducted with the transformed symbionts.

In summary, effector candidates should be anti-pathogenic to the pathogen of interest and have negligible effects on mosquitoes, off-target organisms and symbionts. Undertaking the aforementioned studies would allow the array of potential toxins to be down-selected to generate a library of anti-*Plasmodium* effectors matched with compatible symbionts. These studies have largely been neglected to date but are an important first step for advancing paratransgenesis.

### Could paratransgenesis be used to target arboviruses? Current position

Paratransgenesis to target arboviruses has not been attempted thus far but the antiviral properties of venom toxins are encouraging for this strategy. Antiviral compounds can target various stages of virus infection including pre-entry and/or post-entry stages [52]. Compounds can inactivate the virus pre-entry by inactivating the virus before it attaches to the cell, a process known as neutralisation, inhibiting surface proteins required for attachment, inhibiting virus endocytosis or inhibiting fusion of the viral envelope and host cell membrane. Alternatively, compounds can act at the post-entry stages, by inhibiting viral uncoating, replication, transcription, translation, virus assembly and virus release. Antivirals can also induce the host immune response by stimulating the production of interferons, other cytokines and chemokines, affecting both pre- and post-entry stages. Targeting these stages within the mosquito midgut, as the location of arbovirus infection after the mosquito takes a blood meal from an infected host, may provide the potential to prevent viral dissemination into salivary glands. Blocking this step, as with *Plasmodium*, could prevent the mosquito becoming infectious and transmitting the arbovirus. This approach has been suggested previously as a strategy to control arbovirus transmission [53] but as yet has not been tested.

**Fig. 2:**
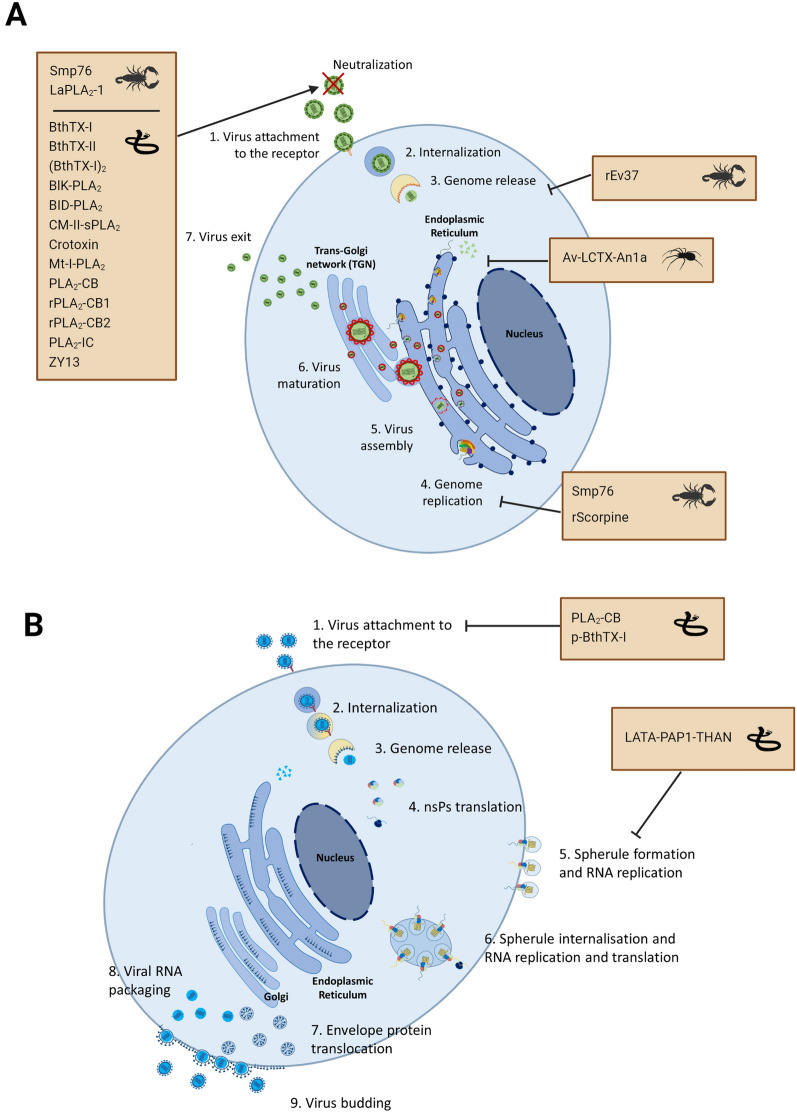
*Orthoflavivirus* (ZIKV, DENV, YFV, JEV) and *Alphavirus* *chikungunya* (CHIKV) stages of infection and venom toxin targets. Venom toxins that have shown antiviral activity against (**A**) *Orthoflavivirus* and (**B**) *Alphavirus* *chikungunya*. The illustration shows the infection stages of the two classes of virus and inhibitory arrows indicate the stage targeted by the venom toxin. Viral particles attach and are internalized through clathrin-mediated endocytosis. Acidification of the endosome facilitates membrane fusion. **A** Once the *Orthoflavivirus* capsid protein is released, the capsid is disassembled and virus genomic RNA transported to the ER for translation and replication. Immature virions bud from the ER and undergo maturation in the trans-Golgi network. Virus exits by exocytosis [69]. **B** After the CHIKV genome release, viral RNA is directly translated into non-structural proteins (nsPs) and forms replication spherules, where viral genome replication occurs. Viral RNA is then translated to produce structural proteins. Capsid proteins and genomic RNA are assembled in the cytoplasm to form icosahedral nucleocapsid, and other structural polyproteins are translocated into ER for post-translational modification and delivered to the cell surface through the secretory pathway. Virus budding occurs when the nucleocapsid assembles with the modified structural proteins [70]. *CHIKV* chikungunya virus, *DENV* dengue virus, *JEV* Japanese encephalitis virus, *YFV* yellow fever virus, *ZIKV* Zika virus

Venom toxins have shown antiviral activity against ZIKV, DENV, YFV, JEV and CHIKV [43, 54–68]. However, there is limited research on the antiviral properties of venom toxins against RVFV. Many antiviral venom toxins have been shown to target the pre-entry stages; the most studied of which are group I and II snake venom PLA_2_ toxins (Fig. 2, Additional file 2: Table S2). Group I PLA_2_ consists of PLA_2_ produced by *Elapidae* (cobras, mambas, coral snakes) and *Hydrophidae* (sea snakes), whilst group II PLA_2_ are produced by *Viperidae* (rattlesnakes) [12]. Group II PLA_2_s derived from *Bothrops* *alteratus* [54]​​, *B.* *leucurus* [55] and *B.* *asper* venom [56] can neutralise several strains of DENV, whilst group II PLA_2_s from *B.* *jararacussu* [57, 58] and *Crotalus* *durissus* *terrificus* venom have shown inhibition activity against YFV, CHIKV, DENV and ZIKV [59–63]. LaPLA_2_-1, a group III PLA_2_ from the scorpion *Liocheles* *australasiae*, can neutralise DENV and JEV [64]. Interestingly, DENV propagated in mosquito cell lines was more sensitive to Mt-I, a catalytically inactive PLA_2_ from *B.* *asper* venom, than viruses propagated in mammalian cells [56]. Neutralisation by group I, II and III PLA_2_ likely occurs by hydrolysis of the virus lipid bilayer [59, 63, 64]. Viral neutralisation has also been shown to occur with ZY13, a peptide analogue of cathelicidin from *Bungarus* *fasciatus* venom [65] and the scorpine-like peptide Smp76 from *Scorpio* *maurus* *palmatus* venom [66, 67].

Venom toxins can also inhibit virus post-entry stages (Fig. 2). Ev37 is a scorpine-like peptide from scorpion *Euscorpiops* *validus* venom that selectively inhibits K_v_1.3 potassium channel and prevents viral genome release into the cytoplasm by acidifying viral genome-containing vesicles, preventing membrane fusion [68]. The host defense peptide Av-LCTX-An1a from *Alopecosa* *nagpag* spider venom can inhibit viral protease activity preventing virus maturation [67]. Studies assessing the host immune response have found that scorpine-like peptide rSmp76 from scorpion *Scorpio* *maurus* *palmatus* venom and ZY13 have antiviral effects by activating interferon signaling [65, 67]. It is important to stress that most of these studies have been undertaken with mammalian cell lines, and their translatability into mosquito cells is unknown. Promisingly, recombinant scorpine generated in *Anopheles* *gambiae* cells can inhibit DENV serotype 2 replication in mosquito cells [43], showing the potential of venom toxins to have antiviral activity within mosquitoes.

### Could paratransgenesis be used to target arboviruses? Future studies

The next step towards evaluating the feasibility of antiviral paratransgenesis would involve identification and validation of appropriate antiviral effectors. This should involve studies with the aforementioned venom toxins to confirm findings and determine whether the antiviral activity seen within mammalian cells is transferable to mosquitoes. Viral neutralization should be assessed by incubating the test compound with the virus and then assessing the virus titre. The effect of the toxins at pre-entry stages should be evaluated by simultaneously adding the toxin and virus to mosquito cells at 4 °C (to prevent virus internalisation) and quantifying the levels of bound virus as well as simultaneously adding the compounds and virus at 37 °C to determine effects on virus internalization and entry. Toxins should also be added after viral infection to evaluate post-entry antiviral activity. The ability of the toxin to induce a cellular antiviral response can be determined by addition of the toxin to the host cells pre-viral infection. In vivo studies assessing viral load, for example by RT-qPCR and plaque assays, in mosquitoes fed with toxins and virus must also be conducted to confirm in vitro findings. Similarly, with antiparasitic effectors, any potential candidates should be further tested as described above to determine any negative effects on mosquitoes, off-target organisms and symbionts. This is a vital step before moving forward with genetically engineering the symbiont to test the plausibility of antiviral paratransgenesis.

## Conclusions

Generation of a paratransgenic approach involves several complex stages, each requiring advanced techniques and considerable time [10]. First, as discussed above, effectors must be identified that inhibit the pathogen without effecting the mosquito, symbiont or off-target organisms through in vitro and in vivo screening [10]. Second, a suitable mosquito symbiont is genetically engineered to appropriately express and secrete an active effector(s), usually by plasmid transformation. The fitness of the symbiont must not be compromised by genetic modification and the symbiont must survive and propagate throughout the mosquito population [10]. Finally, efficacy must be tested within the laboratory and findings confirmed in field trials. This must all be undertaken whilst addressing the safety and public concerns associated with working with and releasing genetically modified organisms [10]. A library of potential effectors would enable researchers to bypass the first stage in this process and venom toxins are excellent candidates to screen.

Venoms contain a highly diverse library of bioactive and stable peptides with antiparasitic and antiviral properties. Studies have shown that using venom toxins as transgenes in paratransgenesis can be useful for controlling mosquito-borne pathogens, specifically *Plasmodium*. However, few studies have screened toxins with the goal of identifying effector molecules [39] and therefore the choice of potential effectors is limited. Here, we have reviewed the toxin literature and have highlighted potential effector candidates for future paratransgenesis studies. However, we stress that additional screening aiming at generating a library of potential effectors is vital. These studies should involve in vitro and in vivo studies to select antiviral and antiparasitic toxins that target appropriate stages of the pathogen life cycle and that do not affect mosquito or symbiont fitness. We also argue the paratransgenesis strategy should be expanded to attempt to target arboviruses. The studies discussed here provide a strong foundation for further research in this area to identify toxin effector candidates.

## Supplementary Information

Additional file 1.

Additional file 2.

## Data Availability

No datasets were generated or analysed during the current study.

## Abbreviations

Arbovirus: Arthropod-borne viruses
CHIKV: Chikungunya virus
DENV: Dengue virus
JEV: Japanese encephalitis virus
mPLA_2_: Inactive mutant (H67N) phospholipase A2
PLA_2_: Phospholipase A2
RVFV: Rift Valley fever phlebovirus
WNV: West Nile virus
YFV: Yellow fever virus
ZIKV: Zika virus
