# Supplementary material for: Exploiting venom toxins in paratransgenesis to prevent mosquito-borne disease
Source: Parasit Vectors. 2025 Jan 29;18:32. doi: 10.1186/s13071-025-06663-9 (PMC11776213; doi:10.1186/s13071-025-06663-9)
Supplement: Supplementary file 1 — Additional file 1. [file 13071_2025_6663_MOESM1_ESM.docx]

| **Supplementary Table 1. Summary of animal venom toxins with antiparasitic activities against the mosquito stages of *Plasmodium*** | | | | | |
| --- | --- | --- | --- | --- | --- |
| **Toxin** | **Origin** | ***Plasmodium* species** | ***Plasmodium* stage** | **Mosquito species** | **Ref** |
| Scorpine | Scorpion *(Pandinus imperator)* | *P. berghei* | Gametocyte to ookinete devlopment | None (*in vitro*) | [43] |
| rMeuTXKβ1 | Scorpion (*Mesobuthus eupeus)* | *P. berghei* | Gametocyte to ookinete devlopment | None (*in vitro*) | [39] |
| Vejovine- and Hadrurin-derived synthetic peptides | Scorpions (*Vejovis mexicanus* & *Hadrurus gertschi)* | *P. berghei* | Ookinete development | None (*in vitro*) | [41] |
| Scorpine | Scorpion (*Pandinus imperator)* | *P. berghei* | Ookinete development | None (*in vitro*) | [42] |
|  |  | *P. falciparum* | Intraerythrocytic stage |  |  |
| VmCT1 | Scorpion (*Vaejovis mexicanus)* | *P. gallinaceum* | Isolated sporozoites | None (*in vitro*) | [40] |
| mPLA_2_ & PLA_2_ | Honey bee (species not stated) | *P. gallinaceum* | Oocyst development in the midgut | *An. fluviatilis* | [45] |
| PLA_2_ | Snake (*Crotalus adamanteus)* | *P. gallinaceum* | Oocyst development in the midgut | *Ae. aegypti* | [44] |
|  |  | *P. falciparum* |  | *An. gambiae* or *An. stephensi* |  |
| Mastoparan X | Wasp (*Vespula lewisii*) | *P.berghei* | Ookinete development *in vitro* | None (*in vitro*) | [38] |
| Melittin | Bee (*Apis mellifera*) | *P.berghei* | Oocyst development in the midgut | *An. stephensi* |  |
| Anoplin | Wasp (*Anoplius samariensis*) | *P. falciparum* |  | *An. gambiae* |  |
