# Supplementary material for: Exploiting venom toxins in paratransgenesis to prevent mosquito-borne disease
Source: Parasit Vectors. 2025 Jan 29;18:32. doi: 10.1186/s13071-025-06663-9 (PMC11776213; doi:10.1186/s13071-025-06663-9)
Supplement: Supplementary file 2 — Additional file 2. [file 13071_2025_6663_MOESM2_ESM.docx]

| **Supplementary Table 2. Summary of animal venom toxins with antiviral activities.** DENV1-4: dengue virus serotype 1-4; YFV: yellow fever virus; ZIKV: Zika virus; CHIKV: chikungunya virus; CHIKV-Nluc: chikungunya virus expressing luciferase; JEV: Japanese encephalitis virus; SINV: Sindbis virus. BthTX-I and BthTX-II, bothropstoxins-I and bothropstoxins-II | | | | | |
| --- | --- | --- | --- | --- | --- |
| **Toxin** | **Origin** | **Virus** | **Cell lines** | **Infection stage(s)** | **Ref** |
| CM-II-sPLA_2_ | Snake *(Naja mossambica mossambica)* | DENV  JEV  SINV | Propagation: Huh7it-1  Infection: Huh7it-1 | Pre-entry | ​​[59]​ |
| BaltPLA_2_ | Snake (*Bothrops alternatus)* | DENV2 | Propagation: C6/36  Infection:  Vero | Pre-entry | ​[50]​ |
| *BI*K-PLA_2_  *BI*D-PLA_2_ | Snake *(Bothrops leucurus)* | DENV1  DENV2  DENV3 | Propagation: C6/36  Infection: LLC-MK2 | Pre-entry | ​​ [51]​ |
| Mt-I PLA _2_ | Snake *(Bothrops asper)* | DENV1  DENV2  DENV3  DENV4  YFV | Propagation: HepG2, C6/36  Infection: BHK-21 | Pre-entry | ​​ [52]​ |
| BthTX-I BthTX-II | Snake *(Bothrops jararacussu)* | ZIKV | Propagation: Vero-E6  Infection: Vero-E6 | Pre- and post-entry | ​​ [53]​ |
| (p-BthTX-I)_2_ | Snake *(Bothrops jararacussu)* | CHIKV-NLuc  ZIKV | Propagation: BHK-21 (CHIKV), C6/36 (ZIKV)  Infection: BHK-21 (CHIKV), Vero (ZIKV) | Pre- and post-entry | ​​ [54]​ |
| crotoxin, PLA_2_-CB, PLA_2_-IC | Snake *(Crotalus durissus terrificus*) | DENV2  YFV | Propagation: C6/36  Infection: Vero-E6 | Pre-entry | ​​ [55,56]​ |
| rPLA_2_-CB1 rPLA_2_-CB2 | Snake *(Crotalus durissus terrificus)* | CHIKV  DENV2  YFV  ZIKV | Propagation: C6/36  Infection: Vero-E6 | Pre-entry | ​​ [57]​ |
| PLA_2_-CB | Snake (*Crotalus durissus terrificus)* | CHIKV | Propagation: BHK-CHIKV-NCT, BHK-21  Infection: BHK-21 | Post-entry | [58] |
| LaPLA_2_-1 | Scorpion (*Liocheles australasiae)* | DENV2  JEV | Propagation: Huh7it-1  Infection: Huh7it-1 | Pre-entry | ​​ [60]​ |
| ZY13 | Snake *(Bungarus fasciatus)* | ZIKV  DENV2 | Propagation: C6/36  Infection: U251, Vero, BMDM | Pre-entry and activates type 1 IFN | ​​ [61]​ |
| Smp76 | Scorpion  *(Scorpio maurus plamatus)* | DENV2 | Propagation: Vero/SLAM  Infection: Vero/SLAM | Pre-entry | [63] |
| rSmp76 | Scorpion (*Scorpio maurus plamatus)* | DENV2  ZIKV | Propagation: Unknown  Infection: Huh-7, THP-1, A549 and PM (DENV);  A549 (ZIKV) | Post-entry and activation type I IFN | [62] |
| rEv37 | Scorpion (*Euscorpiops Validus*) | DENV2  ZIKV | Propagation: Unknown  Infection: Huh7, Vero  293T | Post-entry | [64] |
| rAv-LCTX-An1a | Spider (*Alopecosa nagpag*) | ZIKV  DENV2 | Propagation: Unknown  Infection: HUVEC, A549,  Vero | Post-entry | [67] |
| RScp | Scorpion (*Pandinus imperator)* | DENV2 | Propagation: suckling mouse brain and C6/36  Infection: BHK-21 and C6/36 | Post-entry | [42] |
